# Clinical Effects of a Topically Applied Toll-like Receptor 9 Agonist in Active Moderate-to-Severe Ulcerative Colitis

**DOI:** 10.1093/ecco-jcc/jjw103

**Published:** 2016-05-20

**Authors:** Raja Atreya, Stuart Bloom, Franco Scaldaferri, Viviana Gerardi, Charlotte Admyre, Åsa Karlsson, Thomas Knittel, Jan Kowalski, Milan Lukas, Robert Löfberg, Stephane Nancey, Robert Petryka, Grazyna Rydzewska, Robert Schnabel, Ursula Seidler, Markus F. Neurath, Christopher Hawkey

**Affiliations:** ^a^Medical Clinic 1, Friedrich-Alexander University Erlangen-Nürnberg, Erlangen, Germany; ^b^Gastroenterology, University College London Hospital, London, UK; ^c^Internal Medicine Department/Gastroenterology Division, Catholic University of Rome, Rome, Italy; ^d^InDex Pharmaceuticals, Tomtebodavägen 23A, 171 77 Stockholm, Sweden; ^e^JK Biostatistics AB, Stockholm, Sweden; ^f^Clinical Centre Isacre Lighthouse, IBD Clinical and Research Centre, Prague, Czech Republic; ^g^Stockholm Gastro Center, Sophiahemmet, Stockholm, Sweden; ^h^Department of Medicine, Karolinska Institutet, Solna, Sweden; ^i^Gastroenterology, Lyon-Sud Hospital, Hospices Civils de Lyon, Pierre-Bénite, France; ^j^NZOZ Vivamed, Warsaw, Poland; ^k^Central Clinical Hospital Ministry of Interior in Warsaw, Warsaw, Poland; ^l^Jan Kochanowski University, Kielce, Poland; ^m^Pannonia Maganorvosi Centrum, Budapest, Hungary; ^n^Department of Gastroenterology, Hepatology and Endocrinology, Hannover Medical School, Hannover, Germany; ^o^Department of Gastroenterology, Nottingham Digestive Diseases Centre, Nottingham University Hospitals, Nottingham, UK

**Keywords:** Toll-like receptors, ulcerative colitis, therapy

## Abstract

**Background and Aims:**

Toll-like receptors [TLRs] are potential drug targets for immunomodulation. We determined the safety and efficacy of the TLR-9 agonist DNA-based immunomodulatory sequence 0150 [DIMS0150] in ulcerative colitis [UC] patients refractory to standard therapy.

**Methods:**

In this randomized, double-blind, placebo-controlled trial, 131 patients with moderate-to-severe active UC were randomized to receive two single doses of the oligonucleotide DIMS0150 [30 mg] or placebo administered topically during lower GI endoscopy at baseline and Week 4. The primary endpoint was clinical remission, defined as Clinical Activity Index [CAI] ≤4, at Week 12. Secondary endpoints included mucosal healing and symptomatic remission of key patient-reported outcomes [absence of blood in stool and weekly stool frequency <35].

**Results:**

There was no statistical significant difference between the groups in the induction of clinical remission at Week 12, with 44.4% in the DIMS0150 group vs. 46.5% in the placebo group. However, the proportion of patients who achieved symptomatic remission was 32.1% in the DIMS0150 group vs. 14.0% in the placebo group at Week 4 [*p* = 0.020], and 44.4% vs. 27.9% at Week 8 [*p* = 0.061]. More patients on DIMS0150 compared with those on placebo had mucosal healing [34.6% vs. 18.6%; *p* = 0.09] and histological improvement regarding the Geboes score [30.9% vs. 9.3%; *p* = 0.0073] at Week 4. Significantly more patients on DIMS0150 were in clinical remission with mucosal healing at Week 4: 21% vs. 4.7% in the placebo group [*p* = 0.02]. DIMS0150 was well tolerated, and no safety signals compared with placebo were evident.

**Conclusions:**

Therapy with the topically applied TLR-9 agonist DIMS0150 is a promising and well-tolerated novel therapeutic option for treatment-refractory, chronic active UC patients, warranting further clinical trials.

## 1. Introduction

Ulcerative colitis [UC] is characterized by a superficial, continuous mucosal inflammation, which is predominantly limited to the large intestine.^1–3^ A proportion of patients do not respond to available therapies, thereby becoming treatment refractory and requiring surgical intervention, i.e. proctocolectomy.^4–5^ Therefore, novel treatment strategies are needed. The pathogenesis of UC is poorly understood, but current understanding implies that UC is an immune-mediated condition resulting from the dysregulated balance between commensal enteric flora and the gut-associated immune system.^6^ It is commonly assumed that failure to regulate protective cell-mediated immune responses can result in sustained activation of the mucosal immune system.^7^

One of the ways that a host discerns foreign from self-antigen is through pattern recognition receptors [PRRs], which recognize specific molecular patterns of pathogens.^8^ These include Toll-like receptors [TLRs], which act as innate receptors for pathogen-associated molecular patterns in commensal bacteria. They thus play a pivotal role in mucosal wound healing by controlling the process of epithelial cell proliferation and differentiation.^9^ TLR-9 recognizes exclusively bacterial DNA by serving as a ligand for its CG [CpG] motifs.^10^ These CpG sequence motifs, composed of unmethylated CpG dinucleotides, have been identified as the immunostimulatory component of bacterial DNA.^11^ The ability to modulate both mucosal healing and the immune system makes the CpG motif an attractive therapeutic target. TLR-9 activation has been shown to prevent development of mucosal inflammation and promote wound healing in several models of experimental colitis; however, there was also some controversy in the beginning because some authors proposed a CpG-dependent contribution to the perpetuation of chronic intestinal inflammation.^9,12–16^ In UC patients, a positive correlation between the severity of endoscopic and histological inflammation and TLR-9 expression could be found.^13,17^

DNA-based immunomodulatory sequence 0150 [DIMS0150] [Kappaproct^®^, cobitolimod] is a single-stranded DNA-based synthetic oligodeoxynucleotide [ODN] that contains an unmethylated CpG motif, which activates TLR-9 in target cells such as intestinal T and B lymphocytes and antigen-presenting cells [APCs], with potent induction of anti-inflammatory cytokines such as interleukin-10 [IL-10] and type I interferons.^18,19^ Although administration of DIMS0150 did not meet the pre-specified endpoint in a previous small clinical study in steroid-refractory UC patients, clinical benefit could be observed regarding sustained clinical efficacy.^19^ Similar observations were made in treatment-refractory UC patients within a compassionate use program.^20^

The purpose of this randomized, multicenter, double-blind, placebo-controlled clinical trial was to further define the efficacy and safety of topically administered DIMS0150 in treatment-refractory patients with active UC.

## 2. Material and methods

### 2.1. Patients

This multicenter, randomized, double-blind, placebo-controlled trial [ClinicalTrials.gov identifier: NCT01493960] was conducted at 38 recruiting centers in seven countries [Czech Republic, France, Germany. Hungary, Italy, Poland, and the UK] from December 2011 through March 2014.

Eligible patients were adults with moderate to severely active ulcerative colitis with a Clinical Activity Index [CAI]^21^ of ≥9 and an endoscopic Mayo score of ≥2^22^ [see Table 1], despite treatment with glucocorticosteroids [GCS] [predinisolone equivalent ≥10 mg/day] for ≥2 weeks prior to inclusion, and previously having failed or been intolerant to treatment with mesalazine ≥2.4 g/ day for ≥4 weeks, GCS with at least 0.75 mg/kg as a starting dose, azathioprine or mercaptopurine for ≥3 months, and/or one adequate treatment course of an anti-TNF agent. Patients may have tried treatment with cyclosporine or tacrolimus before the trial. Four patients in the placebo group [9.3%] and 5 patients in the DIMS0150 group [6.2%] had used cyclosporine prior to the study start, but not in the study.

**Table 1.** Demographic and baseline characteristics of patients. Summary of demographic and baseline characteristics of patients assigned into the study [FAS population]. Percentage calculated for the number of subjects by treatment group.

Parameter		Placebo [*n* = 43]	DIMS0150 [*n* = 81]	Overall [*n* = 124]
Age [years]				
*n*		43	81	124
Mean [SD]		43.1 [12.31]	41.1 [13.88]	41.8 [13.34]
Median		43.4	37.7	39.6
Range		23, 69	19, 72	19, 72
Gender, *n* [%]				
Male		32 [74.4]	48 [59.3]	80 [64.5]
Female		11 [25.6]	33 [40.7]	44 [35.5]
Race, *n* [%]				
White		43 [100.0]	79 [97.5]	122 [98.4]
Asian		0 [0.0]	1 [1.2]	1 [0.8]
Black		0 [0.0]	0 [0.0]	0 [0.0]
Other		0 [0.0]	1 [1.2]	1 [0.8]
Weight [kg]				
*n*		43	81	124
Mean [SD]		77.2 [18.56]	72.8 [13.24]	74.3 [15.37]
Median		77.0	73.0	73.7
Range		45, 130	45, 103	45, 130
Current Smoker, *n* [%]				
Yes		3 [7.0]	5 [6.2]	8 [6.5]
No		40 [93.0]	76 [93.8]	116 [93.5]
Past Smoker, *n* [%]				
Yes		15 [34.9]	24 [29.6]	39 [31.5]
No		25 [58.1]	52 [64.2]	77 [62.1]
UC Duration [years]^a^				
*n*		43	81	
Mean [SD]		9.1 [7.5]	9.2 [7.9]	
Median		6.5	6.5	
Range		1.6, 29.6	0.5, 42.8	
CAI score^b^				
*n*		43	81	
Mean [SD]		10.8 [2.03]	11.0 [2.18]	
Median		10.0	10.0	
Range		9, 17	9, 20	
Mucosal appearance [Endoscopic Mayo score]^c^				
Score 0	*n*	0.0	0.0	0.0
	%	0.0	0.0	0.0
Score 1	*n*	0.0	0.0	0.0
	%	0.0	0.0	0.0
Score 2	*n*	17	36	53
	%	39.5	44.4	42.7
Score 3	*n*	26	45	71
	%	60.5	55.6	57.3
Location of inflammation				
Left-sided	*n*	23	56	79
	%	53.5	69.1	63.7
Extensive	*n*	20	25	45
	%	46.5	30.9	36.3
Prior anti-TNF-alpha therapy, *n* [%]				
Yes		17 [39.5]	31[38.5]	48 [38.7]
No		26 [60.5]	50 [61.5]	76 [61.3]
Concomitant medication				
5-ASA or SASP	*n*	32	60	92
	%	74.4	74.1	74.2
Glucocorticosteroids	*n*	43	81	124
	%	100	100	100
Immunosuppressants [Azathioprine, Methotrexate]		12	28	40
	%	27.9	34.5	32.2
TNF-α inhibitors	*n*	0	1	1
	%	0.0	1.2	0.8

^a^Duration is calculated from the date of UC onset to the date of Visit 1.

^b^The last observation carried forward approach was used for missing data.

^c^Mucosal appearance score 0 = normal or inactive [mild granularity, oedema]; score 1 = mild friability, erythema, decreased vascular pattern; score 2 = moderate friability, erosions, marked erythema, absent vascular pattern; score 3 = spontaneous bleeding, ulceration.

SD = standard deviation.

UC = ulcerative colitis.

CAI = clinical activity index.

5-ASA = 5-aminosalicylic acid.

SASP = sulfasalazine.

During the trial, patients could be taking sulphasalazine, aminosalicylates, or thiopurines, and should be on oral GCS at stable doses. Concurrent therapies with cyclosporine, tacrolimus or anti-TNF agents during the trial or in the 4 weeks before enrolment, or antibiotics or non-steroidal anti-inflammatory drugs [NSAIDs] in the 2 weeks before enrolment were not permitted. Patients were excluded if they had a current diagnosis of fulminant colitis, indication for immediate surgery, signs of active infection [temperature ≥38°C], or haemoglobin <100g/L. Additional exclusion criteria were current parenteral nutrition, blood transfusion, *C. difficile* infection, current or past colonic malignancy and/or dysplasia, clinically significant compromise of major organ function, and concurrent or previous use of investigational therapy up to 30 days before enrolment. Women who were pregnant or breast-feeding were excluded.

### 2.2. Study design

**Figure 1.**
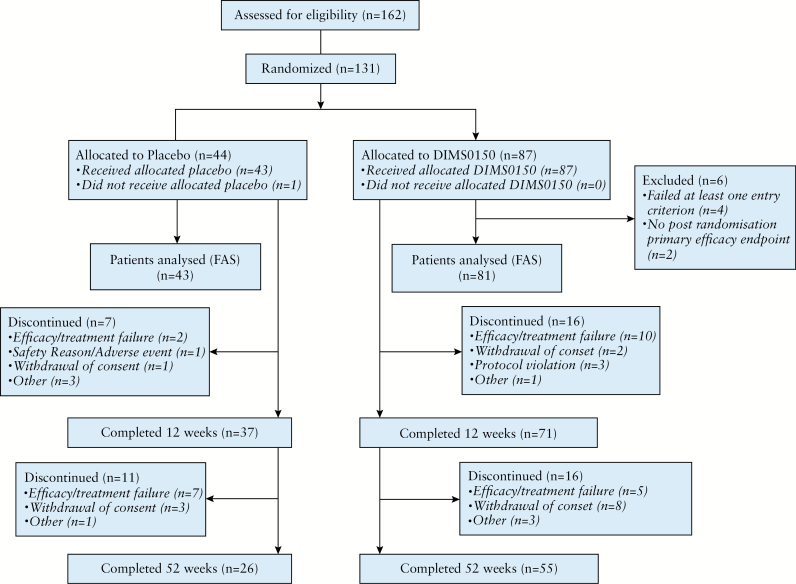
Flow chart of patient disposition in the trial. FAS= full analysis set.

Eligible patients were randomized in a 2:1 ratio to receive administration of DIMS0150 via endoscopy [30 mg] at Week 0 and 4, or matching placebo diluted in 50mL of sterile water after adequate bowel cleaning for stool content. The application was done proximally to the site of mucosal inflammation, or in the transverse section of the colon in the event of extensive colitis, using a spray catheter during endoscopy. Patients were asked to remain recumbent for 2h after administration. Patients were followed up with visits after 1, 4, 8, 12, 22, and 52 weeks [Figure 1]. At Weeks 0, 4, and 12, patients underwent endoscopic evaluation and biopsies were taken from the most inflamed mucosal area. ICON [Texas, USA] produced the randomization code by a computer-generated procedure, which used the method of randomly permuted blocks.

Oral GCS treatment was mandatory at a stable daily dose of ≥10mg initiated at least 2 weeks prior to inclusion. Steroid tapering was done to a standard tapering schedule according to European Crohn’s and Colitis Organisation [ECCO] guidelines^3^ when the patient had reached clinical remission, the earliest at Week 12.

### 2.3. Study drug

DIMS0150 is a fully synthetic 19 mer oligodeoxynucleotide with the sequence 5’-G*G*A*ACA GTT CGT CCA T*G*G*C-3’, where [*] indicates phosphorothioate linkages. The drug substance was manufactured by Avecia [Milford, USA] and the drug product was manufactured by Apoteksbolaget [APL, Umeå, Sweden].

### 2.4. Endpoints

The primary endpoint was induction of clinical remission at Week 12, defined as a CAI score of ≤4. This endpoint was chosen since clinical benefit using this measure was observed in previous compassionate use.^20^ Secondary endpoints included mucosal healing [endoscopic Mayo score ≤ 1, endoscopies not centrally read]; clinical remission at Weeks 1, 4, 8, 22 and 52; clinical remission and mucosal healing; symptomatic remission [defined as absence of blood in stool and weekly stool frequency of <35]; histological Geboes score^23^ at Week 4 and 12 as assessed by a single trial histopathologist; time to colectomy; and quality of life based on the inflammatory bowel disease questionnaire [IBDQ]^24^ and the 36-item short-form survey [SF36] score.^25^ *Post hoc*, the combined score for subjects who achieved both symptomatic remission and mucosal healing was defined.

### 2.5. Safety evaluations

Safety and tolerability were evaluated throughout the study. Safety was assessed by vital signs, ECG results, laboratory variables, and adverse event [AE] reports. Patients were free to discontinue their participation in the study at any time or could be withdrawn from study treatment at the discretion of the investigator.

### 2.6. Statistical analysis

All data were presented using descriptive statistics, with frequency and relative frequency for categorical variables and mean, standard deviation, minimum, and maximum for continuous variables. The power goal in this study was 90% to detect a 45% vs. 15% difference between DIMS0150 and placebo-treated patients, in a 2:1 allocation, with a 0.05 level for type I error, and a two-sided test for hypothesis testing. Calculation was done by the Chi-square test and using the statistical software Nquery 6.0. Sample size calculation revealed that 67 plus 33 subjects were needed, plus 20 subjects [20%] because of dropout. The intention-to-treat principle was used to define the primary analysis population, the Full Analysis Set [FAS], which consisted of all randomized patients that received at least one dose of the study drug [active or placebo], and who had at least one post-randomization eligible value of the efficacy endpoint. The safety population included all patients in the study who received treatment with at least one dose of the study drug. For the efficacy analyses, missing data was replaced using the last-observation-carried-forward [LOCF] method, where appropriate, i.e. for data in the induction phase, 0–12 weeks. The missing data for mucosal healing at Week 8 in the *post hoc* end point of the combined score for subjects who achieved both symptomatic remission and mucosal healing was replaced using last observation carried forward from Week 4 to Week 8.

The primary efficacy endpoint was analyzed by the Chi-square test using the Cochran–Mantel–Haenszel [CMH] method, adjusting for intervals of the CAI score at baseline. The parameter to be tested in the Chi-square test with the CMH was the Odds Ratio [OR]. Secondary categorical efficacy variables were evaluated using the same statistical approach as that described for the primary efficacy endpoint, except for time-to-event endpoints, which were presented using Kaplan–Meier curves and analyzed using the log rank test. The continuous efficacy endpoints of the IBDQ and SF36 subdomains were analyzed using the analysis of covariance (ANCOVA) with regard to the mean change from baseline, and with treatment group and baseline CAI as fixed factors. The statistical hypothesis tested was OE = 1 for the primary endpoint and the secondary categorical endpoints. Further, the hypothesis to be tested did not differ between treatment arms with respect to the mean change from baseline in the continuous efficacy endpoint. The FAS population was used as the primary analysis population. All tests were two-sided, and *p* < 0.05 was regarded as statistically significant. Adjustment for multiplicity was performed for the secondary endpoints, using a hierarchical testing order, starting with induction of clinical remission and symptomatic remission at Week 12.

### 2.7. Approvals

The study protocol was reviewed and approved by regional Independent Ethics Committees and by the competent authorities in each country prior to inclusion of patients [EudraCT nr 2011-003130-14]. Written informed consent for participation in the study was obtained from all patients prior to any study-related procedures.

## 3. Results

### 3.1. Study conduct

A total of 162 patients were screened for inclusion in the study, of which 131 patients were randomized, 87 patients in the DIMS0150 treatment group and 44 patients in the placebo treatment group. One patient in the placebo treatment group was randomized but did not receive treatment [Figure 1]. Of these patients, 61.8% [81/131] completed the study. The timing and reasons for discontinuation are shown in Figure 1. In total, 130 patients were included in the safety analysis population, whereof 87 patients received DIMS0150 and 43 patients received placebo [Figure 1]. The FAS population included 124 subjects: 81 in the DIMS0150 group and 43 in the placebo group [Figure 1].

### 3.2. Patient demographics and disease characteristics

Patients were statistically well matched for demographic data, baseline disease characteristics, mean baseline CAI score, and endoscopic appearances [Table 1].

All FAS patients used GCS: methylprednisolone [36.3%], prednisolone [32.3%], prednisone [32.3%], budesonide [1.6%], or cortisone acetate [0.8%]. The mean prednisolone dose equivalent was 19.7mg [±13.04] for DIMS0150 and 17.7mg [±11.16] for placebo. Most patients had been treated with a previous course of aminosalicylates [74.2%] or thiopurines [78.2%]. Over one-third of the patients [39.5% placebo and 38.5% DIMS0150] had received prior anti-TNF agents, of which infliximab was the most commonly used [81.3%]. Of the patients that had received prior anti-TNF therapy, 75% were treatment refractory. Twenty-two patients [17.7%] had received other immunosuppressive agents [17.3% DIMS0150 and 18.6% placebo]. Fifty percent of the patients in the DIMS0150 group who had received prior treatment with other immunosuppressive agents had stopped these agents due to intolerance [compared with none in the placebo group].

### 3.3. Drug efficacy

#### 3.3.1. Primary and key secondary endpoint

**Figure 2.**
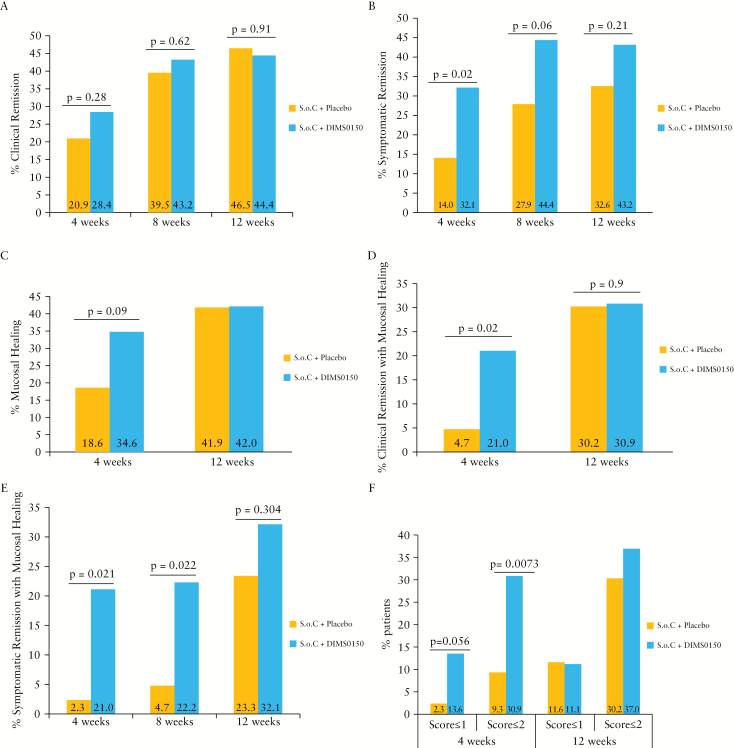
Efficacy of DIMS0150 treatment.

In the FAS population, there were no statistically significant differences between the treatment groups for induction of clinical remission at Week 12, with 44.4% DIMS0150 vs. 46.5% placebo [Figure 2A]. At Week 4, although not statistically significant, the clinical remission rate was higher in the DIMS0150-treated patients compared with the placebo-treated patients, 28.4% vs. 20.9%, respectively [*p =* 0.28].

[A] Proportion of patients with clinical remission defined as clinical activity index [CAI] of ≤4 at 4, 8, and 12 weeks. [B] Proportion of patients with symptomatic remission at 4, 8, and 12 weeks defined as a blood in stool subscore of 0 and a stool frequency subscore of ≤1. Uncombined data on blood in stool and stool frequency are provided as Supplementary Figures. [C] Proportion of patients with mucosal healing at 4 and 12 weeks defined as an endoscopic Mayo score of ≤1. [D] Proportion of patients with clinical remission with mucosal healing at 4 and 12 weeks defined as a CAI score of ≤4 with an endoscopic Mayo score of ≤1. [E] Proportion of patients with symptomatic remission and mucosal healing at 4, 8, and 12 weeks. [F] Proportion of patients with a histological score of ≤2 or ≤1 at 4 and 12 weeks. Last observation carried forward was used for Week 8 endoscopy score. Full analysis set [FAS] was used for the analysis.

In contrast to clinical remission, differences were detected for the secondary endpoint symptomatic remission, i.e. no blood in stool and a stool frequency of <35 per week. The proportion of patients in the FAS population with symptomatic remission at Week 4 was 32.1% for the DIMS0150 group vs. 14.0% for the placebo group [Figure 2B, adjusted OR 3.1, *p =* 0.020]. At Week 8, the proportion of subjects with symptomatic remission was 44.0% for the DIMS0150-treated patients vs. 27.9% for the placebo-treated patients [Figure 2B, OR 2.12, *p =* 0.061]. At Week 12, the proportion of subjects with symptomatic remission was 43.2% for the DIMS0150-treated patients vs. 32.6% for the placebo-treated patients [Figure 2B, OR 1.62, *p =* 0.21]. With respect to mucosal healing, 34.6% of the DIMS0150-treated patients showed mucosal healing [Endoscopic Mayo score ≤1] at Week 4 vs. 18.6% for the placebo-treated patients [adjusted OR = 2.35, *p =* 0.089] [Figure 2C].

Both clinical and endoscopic remission [CAI ≤4 and endoscopic Mayo score ≤1] was seen in 21% of DIMS0150-treated patients vs. 4.7% of patients on placebo [adjusted OR 5.46, 95% confidence interval (CI): 1.15 to 25.86, *p =* 0.0212] at Week 4 [Figure 2D].

#### 3.3.2. Other endpoint analysis

As demonstrated in Figure 2E, the analysis results of the combined score for patients who achieved symptomatic remission and mucosal healing revealed a greater proportion in the DIMS0150-treated group. At Week 4, 21.0% of the DIMS0150-treated patients were in symptomatic remission with mucosal healing vs. 2.3% of the placebo-treated patients [*p =* 0.021]. At Week 8, 22.2% of the DIMS0150-treated patients were in remission compared with 4.7% of the placebo-treated patients [*p =* 0.022]. At Week 12, the proportion of patients in symptomatic remission with mucosal healing was 32.1% in the DIMS0150-treated group and 23.3% in the placebo-treated group [*p =* 0.304].

A *post hoc* analysis examined whether the clinical effects measured by the various endpoints were affected by the site of drug administration—a significant relationship between the site of drug administration and the clinical outcome was not evident.

#### 3.3.3. Histology

At baseline, 38.9% of patients had a histopathological Geboes score of 5, 8.4% a score of 4, 32.1% a score of 3, 10.7% a score of 2, 6.9% a score of 1, and 0% a score of 0, while mucosal appearance was graded as endoscopic Mayo score 3 in 57.3% of patients and as score 2 in 42.7% of patients. At Week 4, a clear improvement in the histological score was observed. A Geboes score of 0–2 was evident in 30.9% of the DIMS0150-treated patients vs. 9.3% of the placebo-treated patients [*p =* 0.0073], and a score of 0–1 [indicating absence of acute inflammation] was evident in 13.6% of the DIMS0150-treated patients vs. 2.3% of the placebo-treated patients [*p =* 0.056] [Figure 2F]. At Week 12, there was no striking difference in the histopathological scoring between the two groups [Figure 2F].

#### 3.3.4. Follow-up evaluations and colectomy rates

The proportion of patients in clinical remission, with a CAI score of ≤4, was similar between the DIMS0150-treated group and the placebo-treated group at 5 months [39.5% vs. 37.2%] and at 12 months [32.1% vs. 32.6%]. The rate of steroid-free clinical remission was 24.7% [*n* = 20] for the DIMS0150-treated group and 23.3% [*n* = 10] for the placebo-treated group at 5 months and 32.1% [*n* = 26] vs. 30.2% [*n* = 13], respectively, at 12 months. Patients randomized to DIMS0150 treatment were able to reduce steroids between Weeks 12 and 52 by 9.8±17.7mg/day prednisolone equivalent vs. an increase for placebo patients of 3.9±24.4mg/day during the same period.

By Month 5, three [3.7%] patients in the DIMS0150-treated group had to undergo colectomy compared with four [9.3%] patients in the placebo-treated group [*p =* 0.121]. By Month 12, four patients [4.9%] of those randomized to DIMS0150 treatment had undergone colectomy compared with five [11.6%] patients in the placebo group [adjusted odds ratio 0.32, CI 0.07–1.37, *p =* 0.116].

#### 3.3.5. Quality of life

There were no significant differences between the treatment groups in quality of life measures, with a reduction in the IBDQ from baseline to Week 12 of 38.6 [CI 29.20 to 48.08] for the DIMS0150-treated group vs. 42.0 [CI 28.27 to 55.71] for the placebo-treated group [*p =* 0.683], data shown in the Supplementary Figures. For the SF-36 general health, the adjusted mean reduction to Week 12 was 3.1 [CI 0.98 to 5.15] for the DIMS0150-treated patients and 6.9 [CI 3.80 to 10.01] for the placebo-treated patients [*p =* 0.04]. There were no obvious differences in sub scores for either index.

### 3.4. Safety evaluations

As shown in Table 2, there were no statistical differences regarding AEs [59.8% of DIMS0150-treated patients vs. 58.1% of placebo-treated patients], treatment-related AEs [11.5% of DIMS0150-treated patients vs. 9.3% of placebo-treated patients], SAEs [11.6% of DIMS0150-treated patients vs. 18.6% of placebo-treated patients] or treatment-related SAEs [3.4% vs. 2.3%] between the groups.

**Table 2.** Summary of safety findings.

	Placebo[*n* = 43]*n* [%]	DIMS0150[*n* = 87]*n* [%]	Overall[*n* = 130]*n* [%]
Patients with AEs^a^	25 [58.1]	52 [59.8]	77 [59.2]
Patients with gastrointestinal AEs^a^	6 [14.0]	16 [18.4]	22 [16.9]
Patients with serious AEs^a^	8 [18.6]	10 [11.6]	18 [13.8]
Deaths	0	0	0
Patients with treatment-related AEs^a^	4 [9.3]	10 [11.5]	14 [10.8]
Patients with treatment-related serious AEs^a^	1 [2.3]	3 [3.4]	4 [3.1]
Patients with AEs^a^ leading to discontinuation	1 [2.3]	2 [2.3]	3 [2.3]

^a^AEs = adverse events.

The most frequently reported AEs were infections [20.8%] and gastrointestinal disorders [16.9%]. The majority of SAEs were assessed as Grade 2 or 3 in severity. The following treatment-emergent SAEs were assessed as Grade 3 severity: overdose of study drug, retinal vein thrombosis, epistaxis, spinal compression fracture, anaemia, and glaucoma. All these events were deemed by the investigator to be not related to the study treatment. Treatment-emergent SAEs that were considered to be treatment-related were reported by four patients [three in the DIMS0150 treatment group and one in the placebo group] and included study drug overdose [one case, DIMS0150-treated], Grade 2 sensory disturbance, and Grade 2 movement disorder on Day 31, 28 days after the last dose of placebo [one patient], an acute coronary syndrome on Day 14, with recovery 2 days later [one case, DIMS0150-treated], and Grade 2 breast dysplasia on Day 78, with recovery on Day 157 [one case, DIMS0150-treated].

## 4. Discussion

In this study, the proportion of UC patients treated with two doses of DIMS0150 at Weeks 0 and 4 who were in clinical remission [primary endpoint], defined by the CAI score ≤4, at Week 12 was not significantly different from that seen in patients treated with placebo. In terms of confirmatory value, the study did therefore not present a positive result. However, these findings are based upon use of an outcome measure that is no longer accepted by regulatory authorities.

By contrast, in an exploratory analysis using criteria that reflect the two items [stool frequency and rectal bleeding] used in the patient-reported outcome 2 [PRO2] score,^26^ DIMS0150 therapy caused statistically significant improvements in symptomatic remission at Weeks 4 and 8 compared with the placebo, with enhanced mucosal healing at Week 4 and statistically significant findings for combined clinical and endoscopic remission at Week 4. These items are the three currently favored items of the Mayo score [stool frequency, rectal bleeding, and endoscopic finding], as they exclude the rather subjective influence of the clinician’s impression of the patient’s disease status [Physicians Global Assessment].^27^ It is very likely that the use of the CAI score to define the primary endpoint in the study was not sensitive enough regarding changes in relevant clinical symptoms, and it excludes indispensable endoscopic assessment.

Another likely reason that the primary endpoint clinical remission was missed is that there was a too long interval between the last treatment dose at Week 4 and the assessment at Week 12. An additional likely contributor was an unexpectedly high rate of clinical remission in the patients treated with placebo, potentially caused by concomitant steroid therapy.

The exploratory analysis also showed that DIMS0150 therapy caused significant improvements in histological disease activity at Week 4 compared with placebo, as assessed by the Geboes score. Athough the Geboes score is a widely used instrument to measure disease activity, this index is not validated, and therefore the observations have certain limitations. In the future this can be overcome by using validated scoring systems for ulcerative colitis, e.g. the Nancy histological index recently published by Marchal-Bressenot *et al.* ^28^

There was some evidence that patients in the DIMS0150 treatment group had a greater reduction in their steroid dose throughout the study compared with the placebo group, thus possibly showing some steroid-sparing effect of DIMS0150. There was also evidence that the rate of colectomy at 5 months was lower in the DIMS0150 group compared with the placebo group, although low patient numbers mean these data must be treated with caution. These considerations require that our secondary and exploratory analyses be given serious consideration, in a limited hypothesis-generating role. They suggest that benefit may be detectable at earlier time points, especially if endoscopic changes are included.

The CAI score contains a large number of outcome measures that are currently considered to introduce subjectivity to the score, or which [in the case of extra-intestinal manifestations] introduce bias because they cannot apply to most patients.^29^ Restricting the symptom analysis to key symptoms [such as bowel frequency and bleeding] that are currently considered reliable and which are similar to those in the Mayo score and PRO-2 assessment, resulted in significant differences between the groups. These analyses provide exploratory evidence for the generation of new hypotheses that DIMS0150 treatment would be superior to placebo if administered according to a different protocol, using measures that are currently regarded as more valid than those that we have used.

Importantly, DIMS0150 treatment was safe and well tolerated. There were no significant SAEs or deaths during the study. The topical application of DIMS0150 clearly adds to the safety profile of DIMS0150, as no systemic uptake of the medication could be observed. In regard to other possible treatment options in refractory UC, systemically given antibodies are associated with multiple AEs [e.g. opportunistic infections and immunogenic reactions], so topical application in UC represents an attractive route of administration regarding therapeutic efficacy with limited systemic AEs.

Among the concepts used to build new therapeutic compounds, the antisense ODN approach is gaining increased attention in the field of IBD. The systemic administration of alicaforsen [targeting intracellular adhesion molecule-1, which mediates the recruitment of leukocytes to the inflamed mucosa] was not effective in Crohn’s disease,^30^ but ameliorated signs of active UC upon topical application.^31^ In a recently performed Phase 2 trial in Crohn’s disease patients, the oral antisense ODN mongersen, which targets the intracellular protein SMAD7 that inhibits transforming growth factor [TGF-]-ß1, demonstrated high efficacy in inducing clinical remission in patients with active disease.^32^

We think the hypothesis that enhancement of mucosal healing and anti-inflammatory properties mediated via TLR9 makes the oligonucleotide DIMS0150 a safe first-in-class therapeutic agent that is worth testing in a future improved trial. As well as basing the primary endpoint upon measures that are currently considered more valid than those used here, such a trial could use a different time from final dose to assessment of the primary endpoint.

In conclusion, therapy with the topically applied TLR-9 agonist DIMS0150 did not reach the defined primary endpoint of induction of clinical remission at Week 12 in active moderate-to-severe UC patients in the present study. However, statistical significant improvements in clinical remission with mucosal healing and symptomatic remission at earlier time points imply that activation of TLR-9 has a therapeutic potential in patients with UC. Subsequent studies are warranted to further identify the therapeutic potential of topically applied DIMS0150 in therapy-refractory patients with chronic active UC, thereby broadening the data regarding the application of ODN in the field of IBD.^33^

## Funding

This study was supported by InDex Pharmaceuticals, Stockholm, Sweden.

## Conflict of Interest

Robert Löfberg, Markus F. Neurath, Åsa Karlsson, Thomas Knittel, Charlotte Admyre, and Jan Kowalski are employees or consultants and/or shareholders of InDex Pharmaceuticals. Raja Atreya, Markus F. Neurath, and Christopher Hawkey are consultants of InDex Pharmaceuticals.

List of Investigators (CSUC-01/10, COLLECT Study Group)

**United Kingdom**: Prof. Chris Hawkey; Dr. Nina Lewis; Dr Stuart Bloom; Dr. Konstantinos Mantzoukis; Dr. Ian Beales, Dr. Mark Tremelling

**Czech Republic**: Dr. Milan Siroky; Dr. Stepan Votocek; Dr. Martin Peterka; Dr. Tomas Vanasek; Prof Milan Lukas; Dr. Martin Bortlik; Dr. Ludek Hrdlicka; Dr. Pavel Klvana; Dr. Pavel Svoboda; Dr. Evzen Machytka; Dr. Milan Kremer; Dr. Jan Smid; Dr. Zdena Zadorova; Dr. Jiri Matous

**France**: Prof. Stéphane Nancey; Dr. Gilles Boschetti

**Germany**: Prof. Daniel Baumgart; Dr. Andreas Fischer; Prof. Jan Konturek; Dr. Ralf Koppermann; Prof. Andreas Stallmach; Dr. Jörg Felber; Dr. Carsten Schmidt; Prof. Ursula Seidler; Dr. Oliver Bachmann; Dr. Clemens Agné; Dr. Christoph Meyer-Heithuis; Dr. Frank Lenze; Dr. Claudia Ott; Dr. Elisabeth Schnoy; Dr. Gisela Paul; Dr. Tamim Lutfi; Prof. Eugen Musch; Dr. Christos Konstantinou; Dr. Peter Hasselblatt; Dr. Michaela Neagu; Dr. Wolgang Kreisel; Dr. Jens Rasenack; Prof. Axel Dignass; Dr. Hermann Schulze; Prof. Heinz Hartmann; Dr. Gisela Felten; Dr. Dietrich Hüppe, Dr. Claudia Mittrop; Dr. Jan Wehkamp; Prof. Stange Eduard; Dr. Simon Jäger; Dr. Oliver Mueller; Dr. Thomas Klag; Prof. Markus Neurath; Prof. Raja Atreya

**Hungary**: Dr. Agnes Salamon; Dr. Ferenc Felföldi; Dr. Beatrix Tam; Prof. Bela Hunyady; Dr. Zoltán Kovács; Dr. Éva Graffits; Dr. Klára Kubinyi; Dr. Robert Schnabel; Dr. Judit Wacha; Dr. Bálint Levente; Dr. Marta Varga; Dr. Klara Csefko; Dr. Timea Pink; Dr. Zsolt Tulassay; Dr. Pál Micheller

**Italy**: Prof. Antonio Gasbarrini; Dr. Franco Scaldaferri; Dr. Viviana Gerardi; Dr. Rodolfo Rocca; Prof. Enrico Corazziari; Dr. Piero Vernia; Dr. Monica Cesarini; Dr. Aurora De Carolis

**Poland:** Prof. Grazyna Rydzewska; Dr. Beata Stepien; Dr. Janusz Milewski; Dr. Kristian Zuk; Dr. Andrzej Bielasik; Dr. Robert Petryka; Dr. Elzbieta Dabrowska-Ufniarz; Dr. Ewa Kucharczyk-Petryka; Dr. Jakub Slowik; Prof Tomasz Mach; Prof. Ewa Malecka-Panas; Dr. Justyna Kotynia; Dr. Lukasz Durko; Dr. Tadeusz Mazurek; Dr. Arkadiusz Mamos; Dr. Izabela Kossowska

## Author Contributions

Robert Löfberg and Christopher Hawkey carried out the conception and design of the study. Raya Atreya, Thomas Knittel, Åsa Karlsson, Jan Kowalski, Charlotte Admyre, and Christopher Hawkey were involved in the conception, design, and analyses of the study, data interpretation, statistical analysis, and drafting and writing of the manuscript. Raja Atreya, Stuart Bloom, Franco Scaldaferri, Viviana Gerardi, Milan Lukas, Robert Petryka, Grazyna Rydzewska, Robert Schnabel, Ursula Seidler, Stephane Nancey, Markus F. Neurath, and Christopher Hawkey were investigators in the COLLECT study. All authors were involved in analysis and interpretation of data, drafting of the manuscript, critical revision of the manuscript, and all authors read and approved the final manuscript. All authors had full access to the data in the study and had final responsibility for the submission to submit for publication.

## Supplementary Data

Supplementary data to this article can be found at *ECCO-JCC* online
